# Supplementary material for: The interactome of intact mitochondria by cross-linking mass spectrometry provides evidence for coexisting respiratory supercomplexes
Source: Mol Cell Proteomics. 2017 Dec 8;17(2):216–32. doi: 10.1074/mcp.RA117.000470 (PMC5795388; doi:10.1074/mcp.RA117.000470)
Supplement: Supplemental Data [file supp_RA117.000470_133922_0_supp_23978_fzffwf.zip › spectra_annotation/mito_spectra_annotation/268-1-19-1-13-1.pdf]

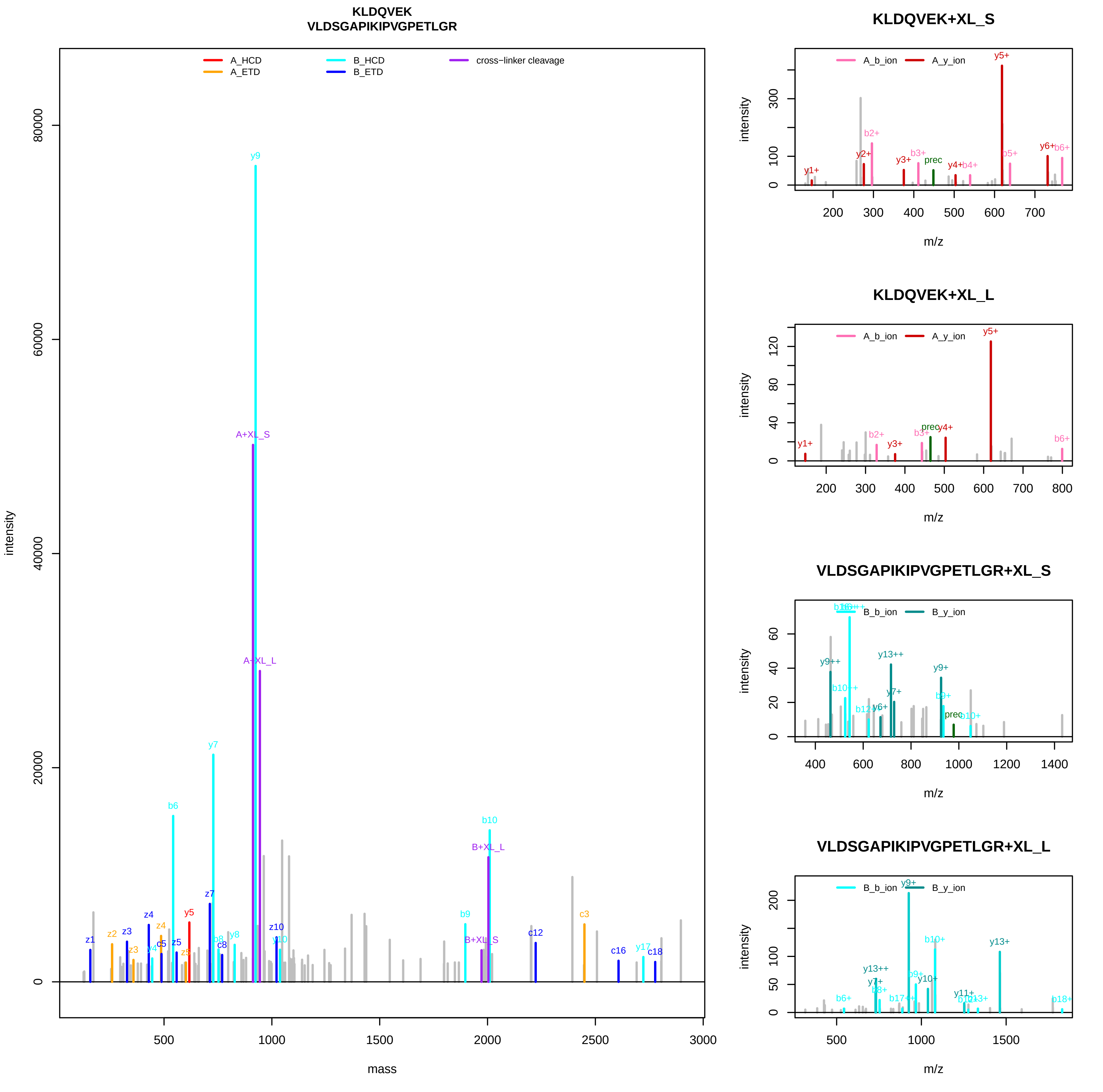
KLDQVEK
VLDSGAPIKIPVGPETLGR
A_HCD
A_ETD
B_HCD
B_ETD
cross−linker cleavage
intensity
mass
y9
A+XL_S
A+XL_L
y7
b6
b10
B+XL_L
B+XL_S
b9
c3
c12
c16
y17
c18
z1
z2
z3
z4
z5
c5
z7
y5
b8
c8
y8
z10
y10
y4
KLDQVEK+XL_S
A_b_ion
A_y_ion
m/z
KLDQVEK+XL_L
VLDSGAPIKIPVGPETLGR+XL_S
B_b_ion
B_y_ion
VLDSGAPIKIPVGPETLGR+XL_L
